# Prognostic impact of rapid reduction of involved free light chains in multiple myeloma patients under first-line treatment with Bendamustine, Prednisone, and Bortezomib (BPV)

**DOI:** 10.1007/s00432-020-03504-3

**Published:** 2021-01-12

**Authors:** Tanja Holzhey, Wolfram Pönisch, Song-Yau Wang, Madlen Holzvogt, Bruno Holzvogt, Marc Andrea, Thomas Zehrfeld, Doreen Hammerschmidt, Franz Albert Hoffmann, Cornelia Becker, Andreas Schwarzer, Maik Schwarz, Uta Schönfelder-Fricke, Thomas Edelmann, Leanthe Braunert, Georg-Nikolaus Franke, Madlen Jentzsch, Sebastian Schwind, Markus Bill, Juliane Grimm, Yvonne Remane, Uwe Platzbecker, Markus Scholz

**Affiliations:** 1 Hematology and Cell Therapy, Medical Clinic and Policlinic 1, University Hospital Leipzig, Liebigstraße 22, 04103 Leipzig, Germany; 2 Hospital Torgau, Christianistraße 1, 04860 Torgau, Germany; 3 HELIOS Vogtland - Klinikum Plauen, Röntgenstraße 2, 08529 Plauen, Germany; 4 Hematology Practice, Biedermannstraße 84, 04277 Leipzig, Germany; 5 Hematology Practice, Strümpellstraße 42, 04289 Leipzig, Germany; 6 Medical Care Center Schöneck, Albertplatz 1, 08261 Schöneck, Germany; 7 Hematology Practice, Lütznerstraße 162, 04179 Leipzig, Germany; 8 Hematology Practice, Theodor - Heuss-Str. 2, 04435 Schkeuditz, Germany; 9 Clinic Pharmacy, University of Leipzig, Liebigstraße 20, 04103 Leipzig, Germany; 10 Institute for Medical Informatics, Statistics and Epidemiology (IMISE), University of Leipzig, Härtelstraße 16-18, 04107 Leipzig, Germany

**Keywords:** Multiple myeloma, Involved free light chain, First-line treatment, Bendamustine, Bortezomib

## Abstract

**Introduction:**

Light chain involvement is observed in almost every patient (pt) with newly diagnosed multiple myeloma (MM). Owing to a relatively short half-life, rapid reduction in the involved free light chain (iFLC) is of potential prognostic value.

**Methods:**

This retrospective analysis included 92 pts with newly diagnosed MM treated with bendamustine, prednisone, and bortezomib (BPV).

**Results:**

After a median number of two (range 1–5) BPV cycles, the majority of pts (*n* = 86; 93%) responded with either sCR (*n* = 21), CR (*n* = 1), nCR (*n* = 25), VGPR (*n* = 20), or PR (*n* = 19). PFS and OS at 48 months were 39% and 67%, respectively. At baseline, 79 out of 92 pts (86%) had iFLC levels above the upper standard level and an abnormal ratio of involved to uninvolved free light chain ≥ 8. In a subgroup analysis of these pts, we evaluated the prognostic importance of an early reduction of the iFLC during the first two BPV cycles. A reduction ≥ 50% of the iFLC on day 8 of the first cycle was observed in 31 of 69 pts. These pts had a significantly better median PFS of 49 months as compared to 20 months in 38 pts with a lower iFLC reduction (*p* = 0.002). In contrast, OS did not differ significantly with a 48 months survival of 77% vs 69% (*p* > 0.05).

**Conclusion:**

These results indicate that a rapid decrease in the iFLC on day 8 is an early prognostic marker for newly diagnosed MM pts undergoing BPV treatment.

## Introduction

Multiple myeloma (MM) is caused by a clonal proliferation of terminally differentiated B cells usually associated with an excessive secretion of monoclonal immunoglobulin (M-protein). In Germany, the incidence of MM is more than eight cases per 100,000 of the population with around 7100 newly diagnosed patients each year (Robert Koch-Institut 2019).

The disease is incurable with conventional chemotherapy (Alexanian and Dimopoulos 1994), with median survival not exceeding 2–3 years (Kumar et al. 2008). The first novel agents introduced into standard therapy were thalidomide, lenalidomide, and bortezomib. These have been followed by many other new drugs (including carfilzomib, daratumumab, elotuzumab, bispecific antibodies, and CAR T cells), which have had a remarkably positive effect on the complete response (CR) rate, progression-free survival (PFS) and overall survival (OS) of MM patients (Kumar et al. 2014; Soekojo et al. 2020). This has resulted in a wide range of therapeutic options, meaning that effective prognostic tools are now required to select the optimal therapy.

Decrease in MM protein is considered as basic prognostic parameter. Almost every symptomatic MM presents with elevated serum free light chain (sFLC) levels at diagnosis where one type, kappa or lambda, dominates (Dispenzieri et al. 2009). Patients with a light chain-predominant secretory MM have an unfavorable clonal evolution leading to a significantly worsened outcome (Brioli et al. 2014; Avivi et al. 2017). Dispenzieri et al. (2008) subsequently examined the baseline involved free light chain (iFLC) value in 399 patients treated between 1988 and 1992 with conventional chemotherapy. In this study, patients were divided into tertiles based on the sFLC concentrations at diagnosis. The patients within the lowest tertile of sFLC (0.3–117 mg/L) showed better outcomes as compared to those within the higher tertiles. Taccetti et al. (2016) analysed the outcome of 110 MM patients treated with a bortezomib-based first-line therapy. A baseline serum free light chain (sFLC) concentration of > 100 mg/L was associated with a lower CR rate and a shorter PFS, but not with a reduced OS. In two other studies, the ratio of sFLC (sFLCr) was identified as an independent prognostic factor in patients treated with conventional chemotherapy only (Kyrtsonis et al. 2007; Snozek et al. 2008). Following the introduction of new drugs, the Spanish PETHEMA/GEM study group investigated the prognostic utility of sFLCr in MM. At diagnosis, a highly abnormal sFLCr was not associated with higher risk of progression (Lopez-Anglada et al. 2018).

Various smaller clinical studies indicate that iFLC analysis can provide additional prognostic information when performed early in a treatment regimen. Normalization of FLCr after one or two therapy cycles was highly predictive for achievement of CR or near CR (nCR) after completion of treatment (Hassoun et al. 2005). In a second study, an early reduction of iFLC was also identified as a predictive factor for better response (≥ very good partial response = VGPR) (Hansen et al. 2014). The mean relative reduction in iFCL three days after start of treatment was 52.3% in patients achieving ≥ VGPR and 23.6% in patients with partial response (PR) (*p* = 0.021). As a predictor of VGPR, an 80% reduction in iFLC at day 21 resulted in a sensitivity of 87.5% and a specificity of 100%. The prognostic effect of sFLC response after two courses of induction therapy was evaluated in two other studies. In the first study, the 5-years OS was 97.4% versus 55.8% in patients with sFLCr ≤ 10 and > 10, respectively (*p* = 0.001) (Yağcı et al. 2015). In contrast, Dispenzieri et al. (2008) found no significant difference in PFS or OS between patients with more or less than 50% reduction of iFLC after two cycles of conventional chemotherapy. In an interim analysis of 303 patients receiving intensive therapy, including a tandem autologous stem cell transplantation (ASCT), during the Total Therapy 3 protocol, both the initial sFLC level and the reduction dynamic were examined (van Rhee et al. 2007). High sFLC levels and/or reduction in response to therapy defined an aggressive MM subtype with poor prognosis. Although the rapid decrease of sFLC on day 7 had no negative effect on the prognosis, a sharp decline after one cycle, or after the first ASCT showed poorer OS.

A disadvantage of these evaluations is the diversity of therapy combinations that were commonly applied in these studies (Dispenzieri et al. 2008; Hansen et al. 2014; Yağcı et al. 2015). Because the rate of myeloma protein reduction likely differs between treatment protocols, studies of single regimes are advantageous to clarify the predictive value. In the present study, we therefore decided to focus exclusively on the combination of bortezomib with bendamustine and corticosteroids as first-line treatment, which has proven to be highly effective in several clinical studies.

In a retrospective analysis, 49 patients with newly diagnosed MM received bendamustine, prednisone, and bortezomib (BPV) (Pönisch et al. 2014). Forty patients (82%) responded after at least one cycle of chemotherapy including 5 stringent CR (sCR), 9 nCR, 12 VGPR, and 14 partial responses (PR). The combination of bendamustine, bortezomib, and corticosteroids was also tested in several phase 2 studies involving newly diagnosed MM patients (Mateos et al. 2015; Berdeja et al. 2017; Knauf et al. 2020). In the study of the Spanish group with 59 newly diagnosed MM patients, the ORR was 84%, including 10% sCR, 14% CR and 33% VGPR (Mateos et al. 2015). PFS at 24 months was 68% for transplant-eligible vs 59% for transplant-ineligible patients, and OS at 24 months was 87% vs 88%. In these studies, responses were rapid, the median time to achievement of PR was one cycle (Pönisch et al. 2014; Mateos et al. 2015).

The results can be further improved by adding high dose melphalan therapy followed by ASCT. After induction with a median of two cycles BPV followed by ASCT as consolidation therapy in 35 patients the ORR was 97% and ≥ VGPR rate 71% (Pönisch et al. 2014).

Therefore, we here consider the BPV protocol as first-line treatment for MM to assess the prognostic value of various laboratory parameters particularly focusing on early reductions of iFLC.

## Methods

### Patients

Patients with newly diagnosed MM treated in the Hematological Department of the Medical Clinic and Policlinic 1 of the University Hospital Leipzig with BPV between January 2013 and October 2019 were included in this retrospective analysis. All patients met CRAB criteria (Rajkumar et al. 2014) or had another myeloma-related end-organ damage. Both transplant-eligible and transplant-ineligible patients were included in this investigation. Patients with nonsecretory MM, neuropathy grade ≥ 3 or active nonhematological malignancy during the past 3 years were excluded. All patients gave written informed consent for the applied treatment and the use of anonymised personal data for clinical research. The study was approved by the Ethical Committee at the Medical Faculty, Leipzig University (IRB 00001750; registration number 118/18-ek).

### Treatment protocol

Bendamustine (60 mg/m^2^) was given as a 30-min infusion on days 1 and 2 in combination with oral prednisone (100 mg) on days 1, 2, 4, 8, and 11, and bortezomib (1.3 mg/m^2^) as an intravenous push or subcutaneously on days 1, 4, 8, and 11 (Pönisch et al. 2012). In dialysis-dependent patients, bendamustine and bortezomib were given 30 min after dialysis. In transplant-ineligible patients, BPV cycles were repeated every 21 days for up to five cycles until a maximum response or disease progression was observed. The maximum response was achieved if three additional weeks of therapy did not result in a further reduction of myeloma protein by more than 10% in serum and/or urine. In transplant-eligible patients, the therapy was stopped prematurely after a minimum of two BPV cycles if PR was achieved.

### Definitions of response

Evaluation of response was based on the *international uniform response criteria for multiple myeloma* (Durie et al. 2006). In addition, the terms ‘near complete response’ (nCR) and ‘minor response’ (MR) were considered.

Response data were collected on day 1, 8, 15, and 22 of each BPV cycle. OS, respectively, PFS were determined as time difference between start of therapy and time at censoring or death, respectively relapse, progression, or death.

### Evaluation of efficacy

Patients were examined within 48 h prior to inclusion into the BPV protocol. Staging was performed for each patient comprising medical history, physical examination including a detailed neurological examination, determination of *World Health Organization Performance Status*, determination of laboratory parameters (including β_2_-microglobulin, serum protein, serum protein electrophoresis, myeloma typing of serum and urine, serum free light chain assay (Freelite®), serum creatinine, serum calcium, and C-reactive protein), electrocardiogram, low-dose CT, and bone marrow biopsy. Myeloma protein concentration was determined by the integral of the area under the myeloma protein curve (based on electrophoresis data) and by relating it to the total serum protein. Renal function was assessed by the eGFR using the Modification of Diet in Renal Disease (MDRD) formula (Levey et al. 1999). Patients were followed-up at 4-weekly intervals during the first three months after the end of treatment, and thereafter, at 12-weekly intervals until disease progression.

### Statistical methods

Descriptive statistics were calculated for demographic and baseline variables. Regarding the survival follow-up, the data set was freezed at 31st of October 2019. All patients commenced to treatment until this date were included in the analysis. OS and PFS were estimated by the Kaplan–Meier method. Survival curves are compared by Log Rank tests (IBM SPSS Statistics, Version 24). *P* values of group differences were calculated applying the Wilcoxon rank sum test. Categorical variables were compared using the *χ*^2^ test. *P* values < 0.05 were considered statistically significant.

## Results

### Patient characteristics

92 patients with newly diagnosed/untreated MM were enrolled in this retrospective analysis. All patients completed at least one cycle of BPV therapy and were therefore available for analysis. Baseline demographics and disease characteristics are shown in Table 1. Patients were divided into three groups differing in their myeloma class: IgG myeloma (*n* = 42), IgA myeloma (*n* = 23) and other myeloma (IgD, *n* = 1; light chain, *n* = 26). Kappa light chains were affected in 54 patients (59%) and lambda light chains in 38 patients (41%).

**Table 1:** Baseline characteristics of the 92 patients with newly diagnosed/untreated MM

Parameter	IgG MM (*n* = 42)	IgA MM (*n* = 23)	Other MMIgD (*n* = 1) Light chain (*n* = 26)
Median age, years (range)	61 (36–88)	62 (41–76)	61 (46–71)
Male, *n* (%)	25 (60)	15 (65)	18 (67)
ECOG PS (*n* (%))			
0	0	0	0
1	12 (29)	9 (39)	2 (7)
2	22 (52)	7 (30)	18 (67)
3	7 (17)	6 (26)	7 (26)
4	1 (2)	1 (4)	0
Type of light chain (*n* (%))			
*Κ*	28 (67)	10 (44)	16 (59)
Λ	14 (33)	13 (56)	11 (41)
iFLC (*n* = 79)^a^	32 (76)	20 (87)	27 (100)
eGFR (ml/min, *n* (%))			
≥ 60	27 (64)	16 (70)	13 (48)
30–59	6 (14)	1 (4)	6 (22)
15–29	4 (10)	3 (13)	5 (19)
< 15	5 (12)	3 (13)	3 (11)
Durie–Salmon stage (*n* (%))			
Ia	1 (2)	0	1 (2)
Ib	2 (5)	0	0
IIa	1 (2)	1 (4)	1 (2)
IIb	1 (2)	0	0
IIIa	30 (71)	16 (70)	16 (59)
IIIb	7 (17)	6 (26)	9 (33)
ISS stage (*n* (%))			
I	16 (38)	8 (35)	4 (15)
II	13 (31)	6 (26)	9 (33)
III	13 (31)	9 (39)	14 (52)
rISS stage (*n* (%))			
I	10 (24)	2 (9)	2 (7)
II	13 (31)	6 (26)	9 (33)
III	19 (45)	15 (65)	16 (60)
Genetic risk (FISH)^b^			
Normal risk	27(73)	10 (43)	18 (72)
High risk^c^	10(27)	13 (57)	7 (28)

^a^Involved free light chain level above the upper standard level and an abnormal free light chain ratio of involved to uninvolved free light chain ≥ 8

^b^Results available from 85 patients (IgG MM: *n* = 37; IgA MM: *n* = 23; other MM: *n* = 25)

^c^High risk: del(17p), *t*(4;14), *t*(14;16), *t*(16;20)

Median age was 61 (range 36–88) years. The analysis included 22 patients (24%) with a poor general condition (ECOG performance status grade 3/4). Two patients with Salmon and Durie stage Ia, one patient with multilocular myeloma cell infiltration of the skin and a second patient with cardiac amyloidosis, were also included in the analyses. Twenty-three patients (25%) had a severe renal failure (eGFR < 30 ml/min), and seven of these required dialysis (8%). Moderate, short-term hypercalcemia (2.61–2.93 mmol/L) was observed in ten patients (11%) and severe hypercalcemia (3.17–4.0 mmol/L) in five patients (5%). Chromosomal aberrations were examined with interphase fluorescence in situ hybridization (FISH) in 85 patients. 30 patients (35%) had at least one high-risk cytogenetic abnormality: del17p (*n* = 14; 16%), *t*(4;14) (*n* = 14; 16%); *t*(14;16) (*n* = 5; 6%).

### Response and survival

The majority of patients (*n* = 81; 88%) responded after a median of two (range 1–5) BPV cycles with 5 sCR (5%), 11 nCR (12%), 25 VGPR (27%), and 40 PR (43%) (Table 2).

**Table 2:** Number of treatment cycles and outcome following treatment with bendamustine, prednisone, and bortezomib in 92 patients with newly diagnosed/untreated MM

Parameter	IgG MM (*n* = 42)	IgA MM (*n* = 23)	Other MM IgD (*n* = 1) Light chain (*n* = 26)
Number of cycles, median (range)	2 (1–4)	2 (2–5)	2 (1–5)
Number of cycles to first response, median (range)	1.3 (0.33–3)	1 (0.33–2)	0.33 (0.33–1.67)
Number of cycles to maximum response, median (range)	2 (1–4)	2 (1–3)	1 (1–3)
ASCT as consolidation therapy, n (%)	25 (60)	18 (78)	22 (82)
Progression free survival, median (months)	39	24	35
Overall survival 4 years (%)	72	64	69

The treatment was continued until achievement of maximum response in 27 nontransplant-eligible patients. Sixty-five patients discontinued BPV therapy prematurely after a median of two cycles to receive autologous (*n* = 58) or autologous/allogeneic SCT (*n* = 7). After SCT as consolidation therapy, the ORR in all patients increased to 93% (*n* = 86) with 21 sCR (23%), 1 CR (1%), 25 nCR (27%), 20 VGPR (22%), and 19 PR (21%) (Table 3). The BPV regime leads to a rapid decrease in myeloma protein, with 25 (27%) patients reaching their best response after the first cycle and an additional 41 patients (45%) after their second cycle. The median time to first hematological response (≥ PR) was 21 days.

**Table 3:** Best confirmed hematological response to treatment with bendamustine, prednisone and bortezomib in 92 patients with newly diagnosed/untreated MM (including ASCT as consolidation therapy in 65 patients)

Best confirmed response	No. of patients (%)		
	IgG MM (*n* = 42)	IgA MM (*n* = 23)	Other MM IgD (*n* = 1) Light chain (*n* = 26)
Stringent complete response	4 (10)	9 (39)	8 (30)
Complete response	1 (2)	0	0
Near complete response	10 (24)	10 (44)	5 (19)
Very good partial response	10 (24)	3 (13)	7 (26)
* ≥ VGPR*	*25 (60)*	*22 (96)*	*20 (74)*
Partial response	13 (31)	1 (4)	5 (19)
* ≥ PR*	*37 (90)*	*23 (100)*	*25 (93)*
Minor response	2 (5)	0	0
Stable disease	2 (5)	0	2 (7)
Progressive disease	0	0	0

**Fig. 1:**
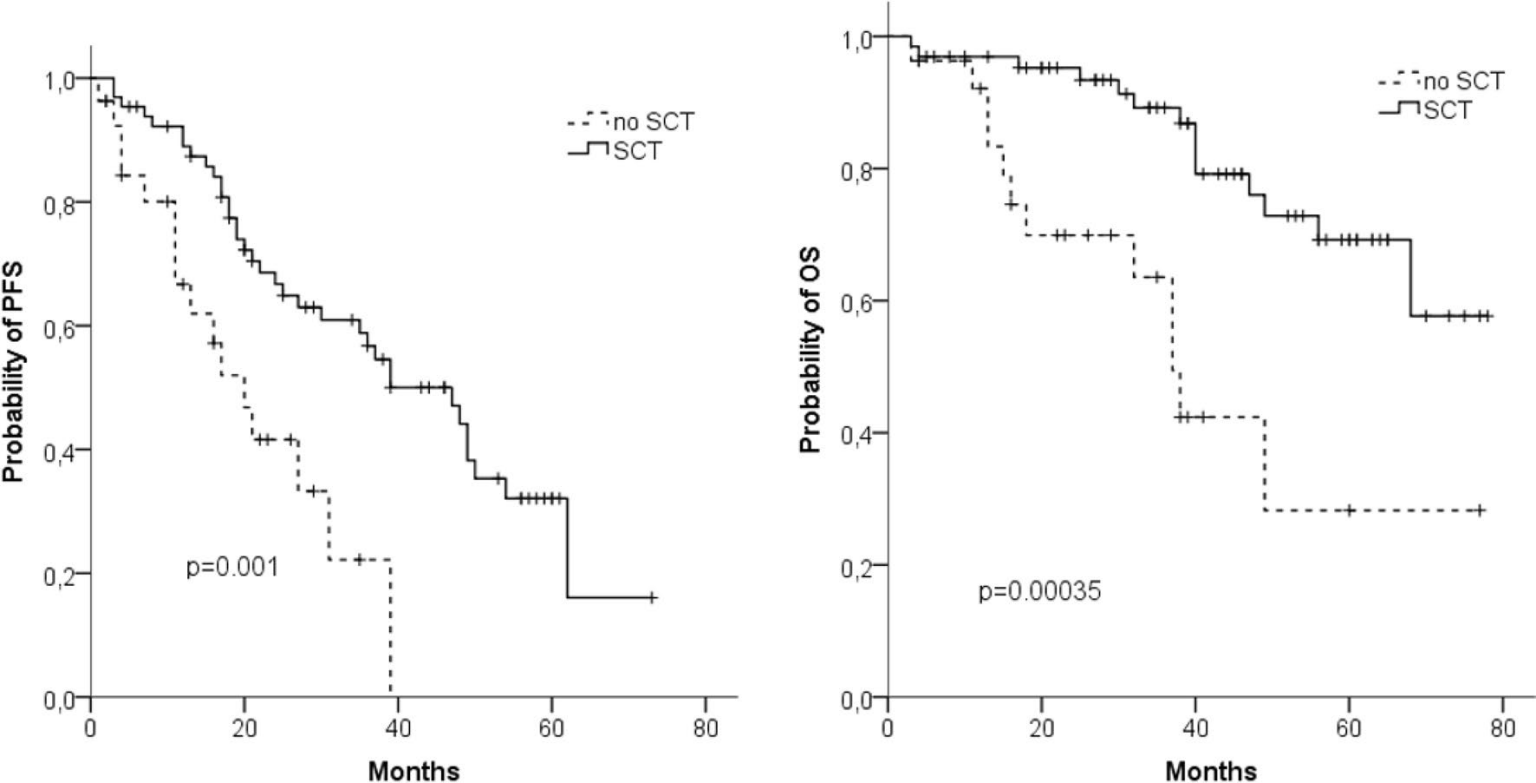
Progression-free survival (PFS) (**a**) and overall survival (OS) (**b**) in transplant-eligible (*n* = 65) and non-eligible (*n* = 27) patients

After a median observation time of the surviving patients of 37 months, 48 months PFS was 39% and 48 months OS was 67%. The median PFS of transplant-eligible patients (*n* = 65) was 37 months and the median OS was not reached (Fig. 1a, b). Outcome for these patients was significantly better as compared to patients not eligible for SCT (*n* = 27) with a median PFS and OS of 20 months and 37 months, respectively.

### Prognostic factors

The 48-month PFS was 57% in patients with ISS stage I, 52% in stage II, and 16% in stage III while OS at 48 months was 82%, 60%, and 56%, respectively. Although the difference in PFS between patients in stage I and II compared to stage III was significant (*p* = 0.004) there were no significant differences in OS (*p* = 0.34).

**Fig. 2:**
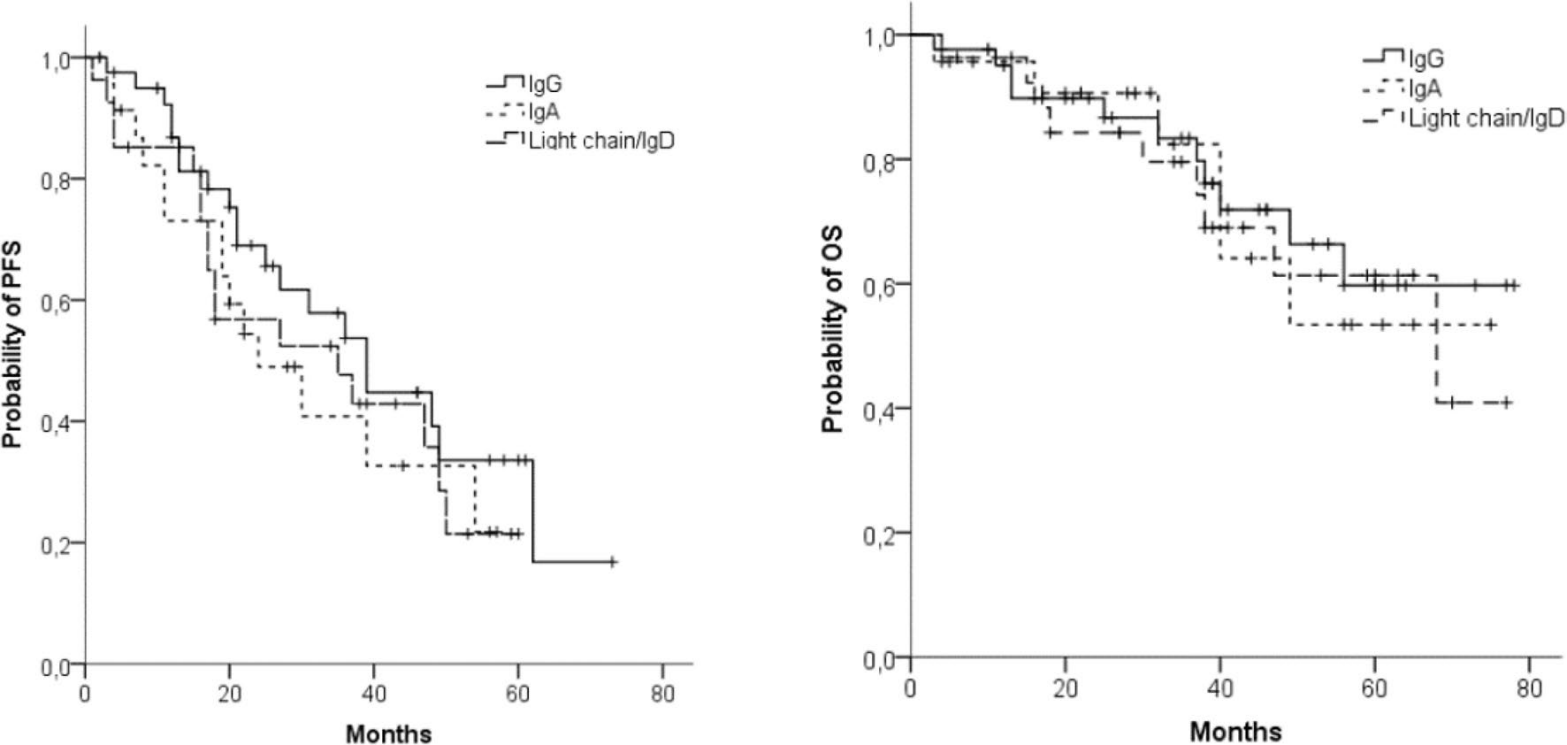
Progression-free survival (PFS) (**a**) and overall survival (OS) (**b**) in different MM subclasses: IgG (*n* = 42), IgA (*n* = 23), light chain/IgD (*n* = 27)

We found no relevant difference in ORR between patients with IgG MM (*n* = 37; 90%), IgA MM (*n* = 23; 100%), and IgD/light chain MM (*n* = 25; 93%). However, the number of sCR/CR was significantly higher in patients with IgA MM (*n* = 9; 39%) and IgD/light chain MM (*n* = 8; 30%) than in patients with IgG MM (*n* = 5; 12%, *p* < 0.03). There were no significant differences in PFS and OS between the different MM classes (Fig. 2a, b).

**Fig. 3:**
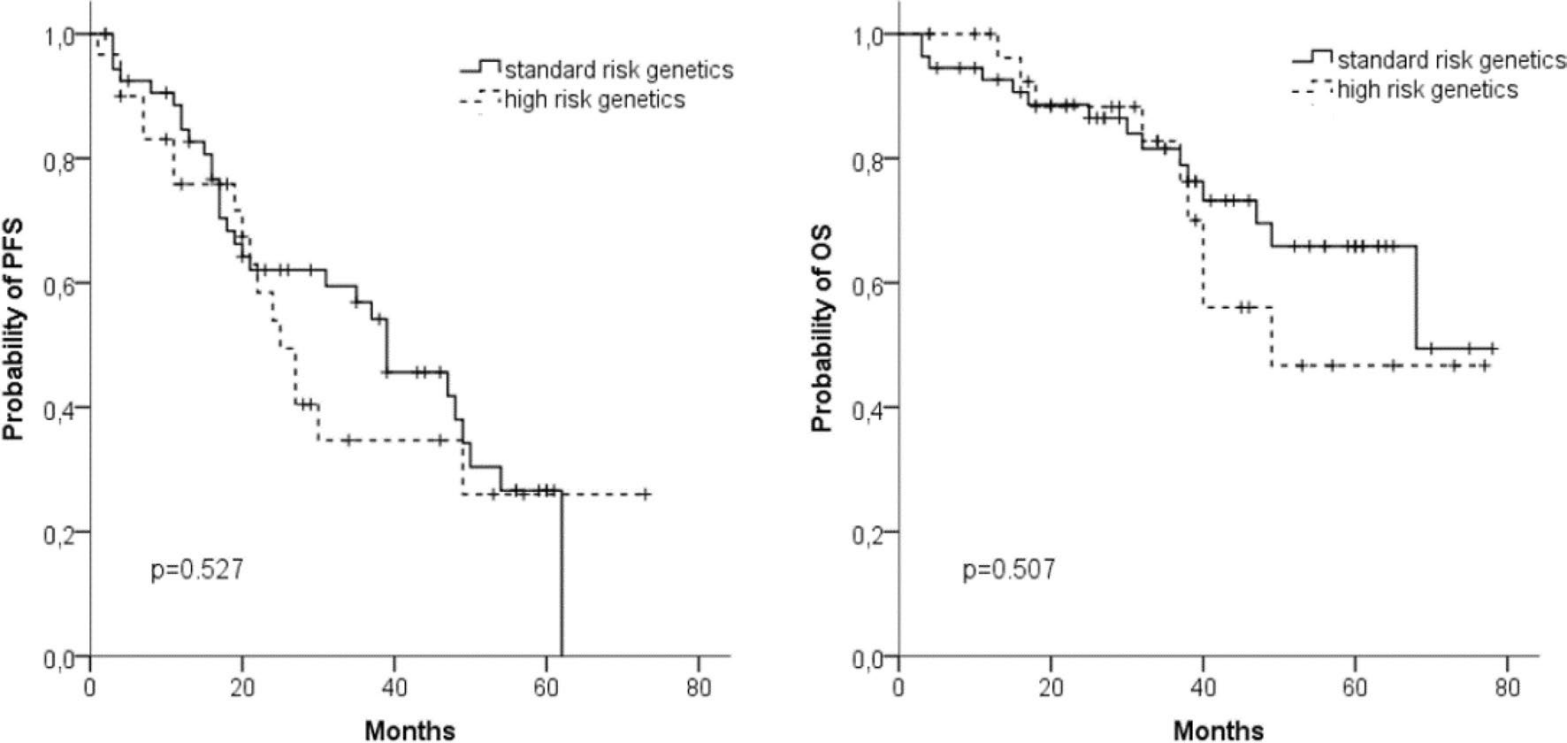
Progression-free survival (PFS) (**a**) and overall survival (OS) (**b**) in patients with (*n* = 30) or without (*n* = 55) high-risk cytogenetics (*t*(4;14), *t*(14;16), del(17p))

We analyzed the prognostic impact of chromosomal aberrations in 85 patients. In patients with high risk cytogenetics (*n* = 30), we found no differences in median PFS (25 vs 39 months) and median OS (49 vs 68 months) compared to patients with standard risk (*n* = 55) (Fig. 3a, b).

**Fig. 4:**
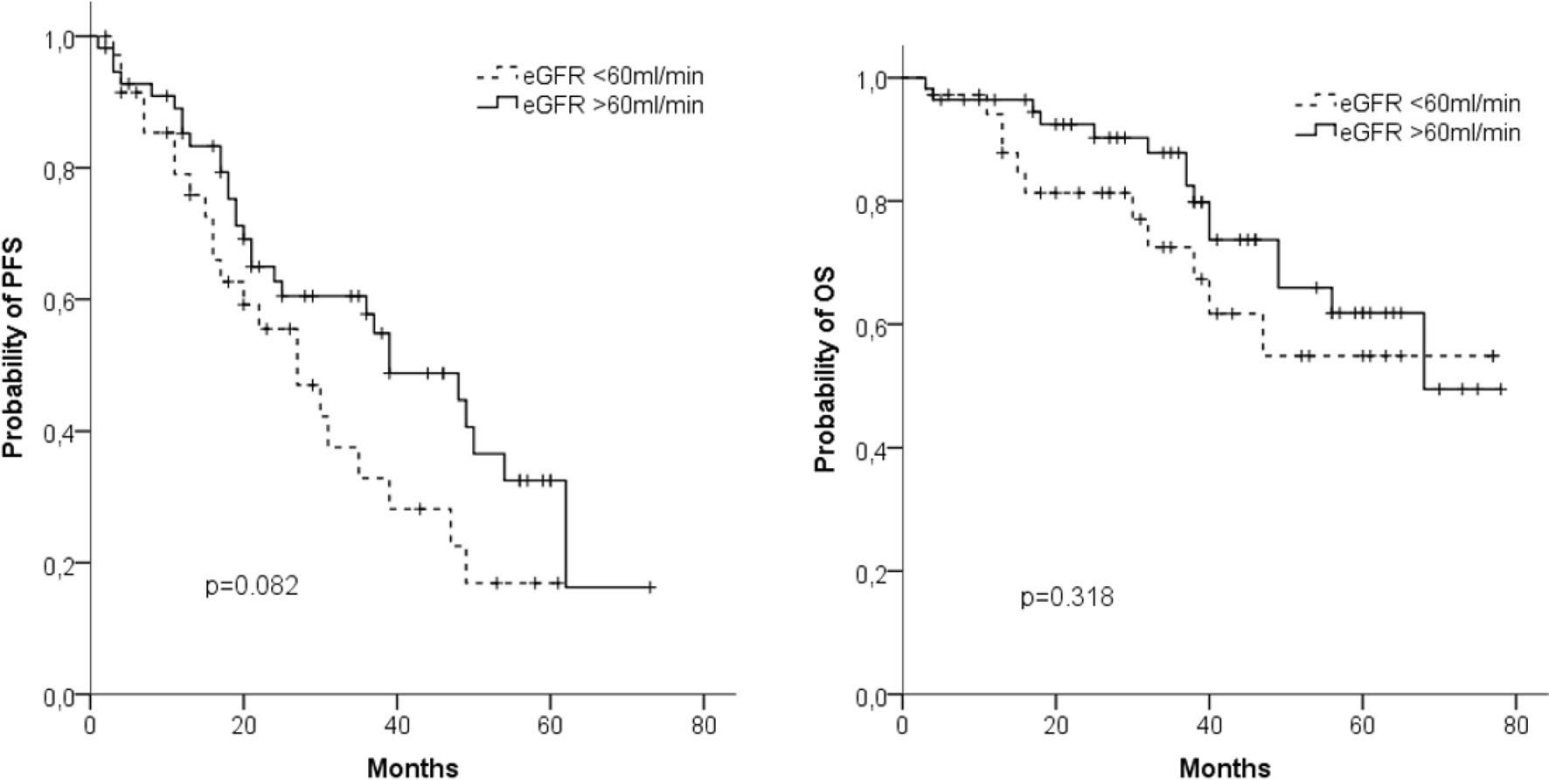
Progression-free survival (PFS) (**a**) and overall survival (OS) (**b**) in patients with GFR < 60 ml/min (*n* = 56) and those with (*n* = 36) GFR ≥ 60 ml/min

Regarding renal function, we found no differences between patients with normal function or mild dysfunction (eGFR ≥ 60 ml/min) on the one hand and patients with moderate or severe renal dysfunction (eGFR < 60 ml/min) on the other hand (median OS: 68 months vs nr; median PFS 39 vs 27 months) (Fig. 4a, b). Altogether, 23/36 (64%) patients with impaired eGFR experienced an improvement in their renal function. Fifteen patients reached a CRrenal, two a PRrenal and six an MRrenal. Three of the seven dialysis patients became dialysis independent.

### Prognostic impact of free light chain ratio and early involved free light chain reduction

**Fig. 5:**
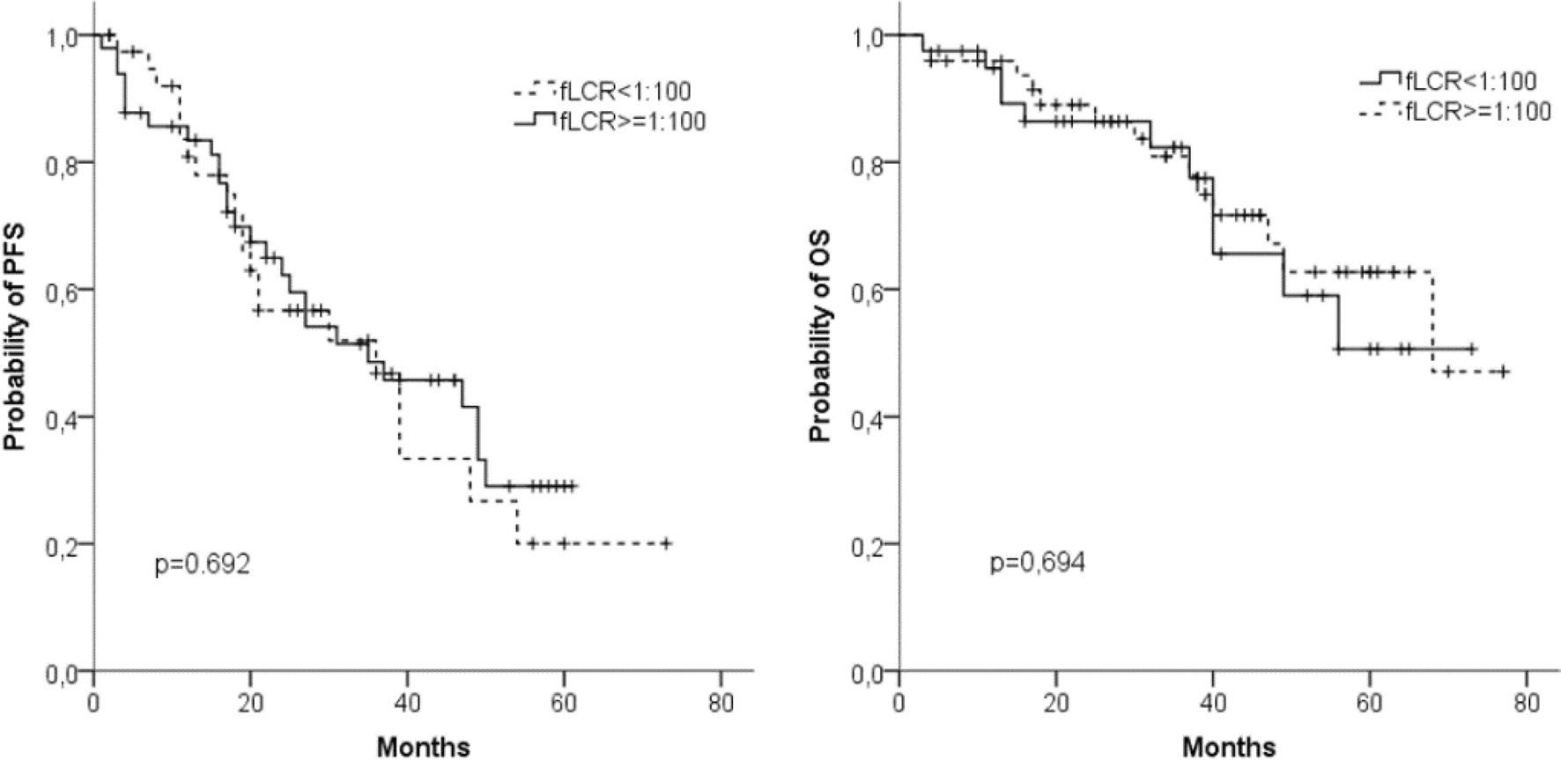
Progression-free survival (PFS) (**a**) and overall survival (OS) (**b**) in 89 patients with involved to uninvolved free light chain ratio (iFLCr) > 100 (*n* = 49) or ≤ 100 (*n* = 40)

At the time of diagnosis, FLC data were available for 89 patients. 49 (55%) of these had a ratio of sFLCs < 0.01 or > 100 (Fig. 5a, b). Comparison with the other 40 patients (45%) revealed no difference in PFS or OS. We also observed similar median PFS (31 vs. 39 months) and median OS (n.r. vs. 68 months) in patients with sFLC ≥ 750 mg/L (*n* = 41) and < 750 mg/L (*n* = 48).

79 out of 89 patients (89%) had an iFLC level above the upper standard and an abnormal ratio of involved to uninvolved FLC ≥ 8: 32 out of 42 patients with IgG MM, 20 out of 23 with IgA MM, and all 27 with light chain/IgD MM (Table 1). In a subgroup analysis of these patients we evaluated the prognostic importance of an early reduction of the iFLC during the first two BPV cycles (Table 4).

**Table 4:** Prognostic impact of an early reduction of the iFLC during the first two BPV cycles in 79 patients

	iFLC reduction (%)	Patients^a^ (*n*)	Ratio^b^	PFS Median (month)	*p* (log rank)	OS Median (month)	*p* (log rank)
Day 8	≥ 50	69	31 vs 38	49 vs 20	0.002	68 vs nr	0.126
Day 15	≥ 75	58	29 vs 29	37 vs 25	0.184	68 vs nr	0.381
Day 22	≥ 75	74	38 vs 36	47 vs 35	0.204	nr vs 68	0.253
Day 43	≥ 75	71	51 vs 20	47 vs 36	0.486	nr vs 56	0.328

*nr* not reached

^a^Number of patients examined on that day

^b^Patients with higher iFLC reduction compared with patients with lower iFLC reduction

**Fig. 6:**
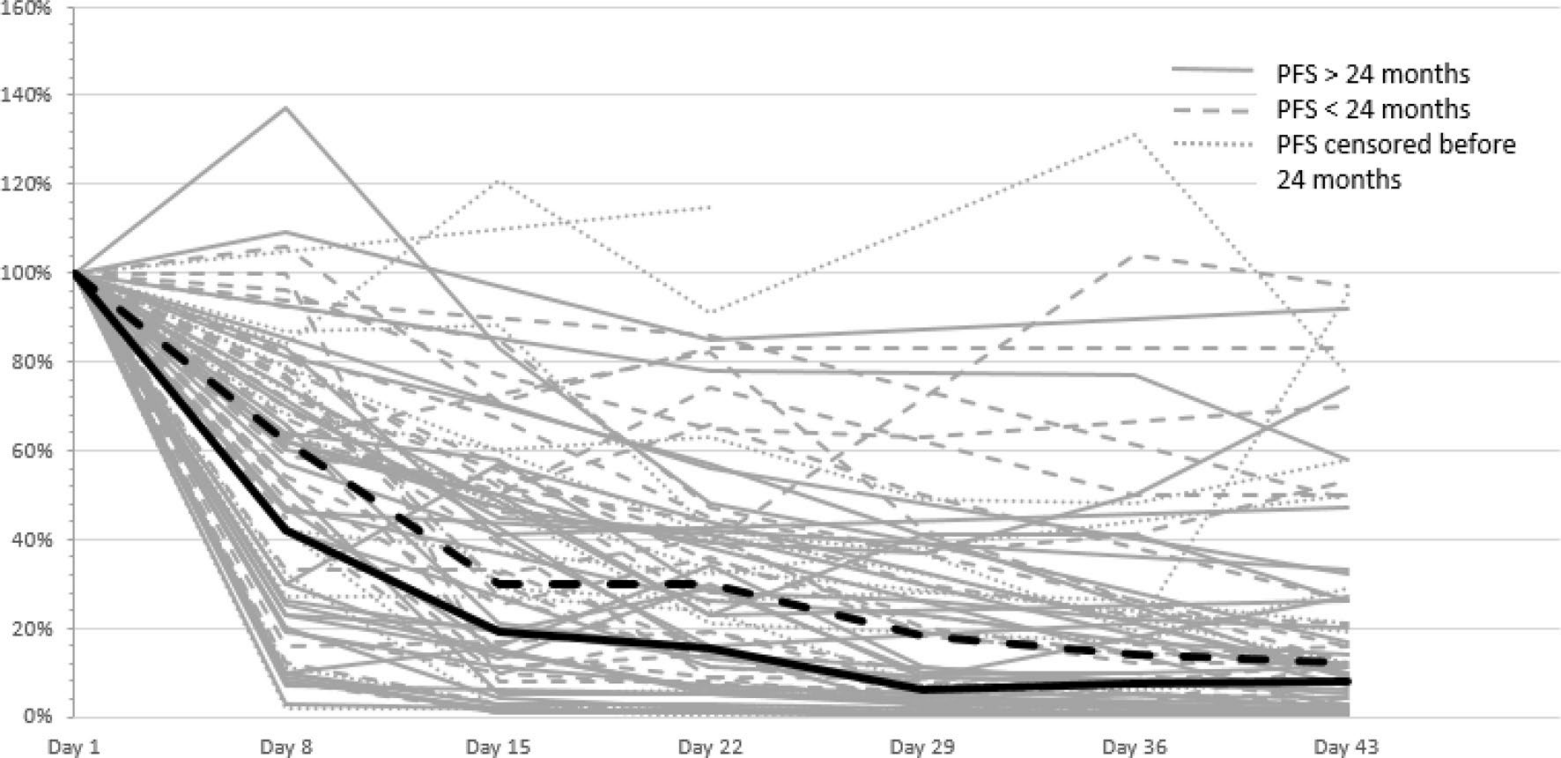
Kinetics of involved free light chains during first two BPV cycles. Continuous line: patients with PFS > 24 months (*n* = 35), dashed line: patients with PFS < 24 months (*n* = 28), dotted line: patients with censored PFS within the first 24 months (*n* = 16). Thick lines representing median reduction in the first both groups

The individual curves of declining iFLCs in the first two treatment cycles are shown in Fig. 6. Only on day 8, we observed a significant difference of the median reduction of iFLC between patients with a PFS ≥ 24 months and < 24 months (58% vs. 38%, *p* < 0.05). This led us to the conclusion that day 8 is of special interest to prognosis.

**Fig. 7:**
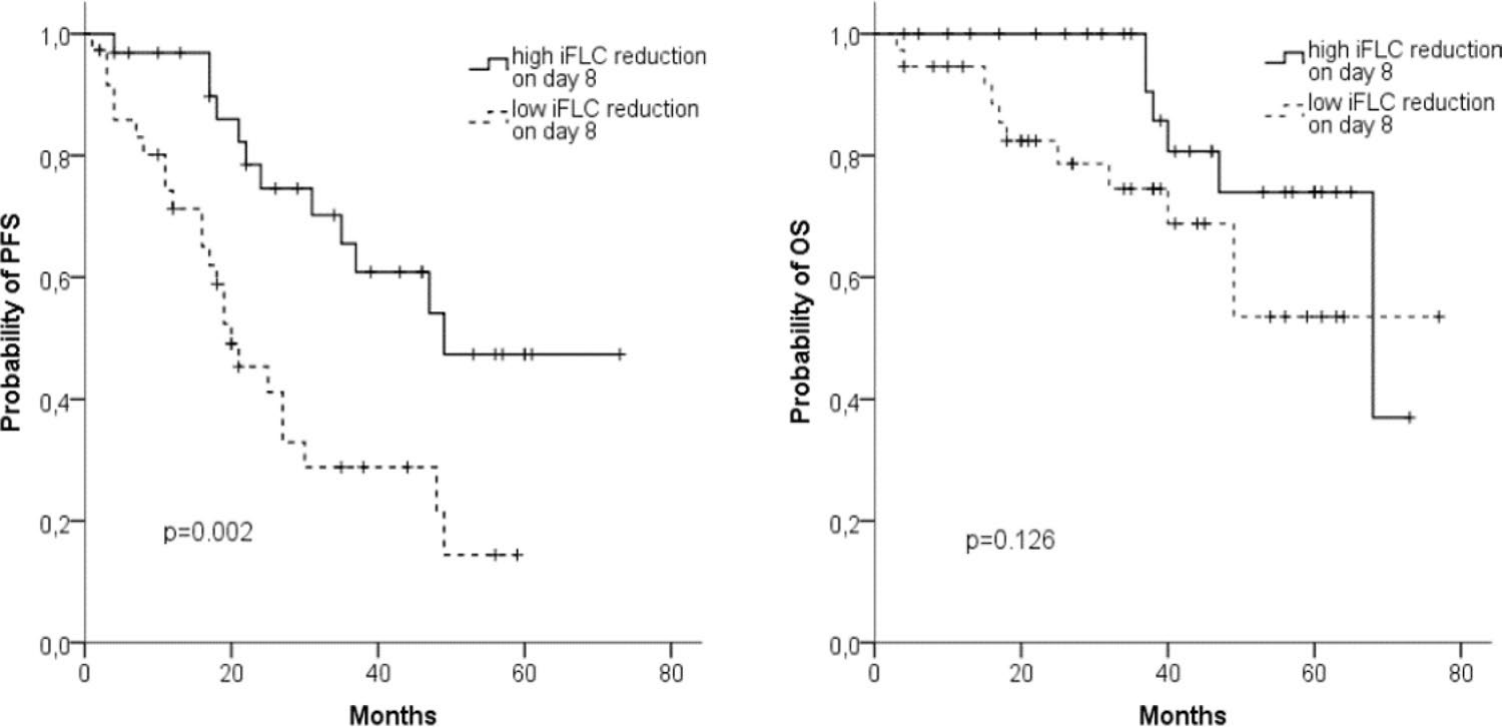
Progression-free survival (PFS) (**a**) and overall survival (OS) (**b**) in 69 patients with a reduction of ≥ 50% (*n* = 31) of the involved free light chain on day 8 or < 50% (*n* = 38)

A reduction ≥ 50% of the iFLC on this day was identified in 31 of 69 patients. These patients had a significantly better median PFS of 49 months compared to 20 months in the 38 patients with a lower iFLC reduction (Fig. 7a). In contrast, OS did not differ significantly with a 48 months survival of 77% vs 69% (Fig. 7b). There was also no difference in the ORR with 31/31 (100%) vs 33/38 (87%) patients in the two groups. However, the number of patients attaining CR/sCR was markedly and significantly larger in the group who experienced a rapid decrease in iFLC (14 patients, 45%) compared to the group who did not (4 patients, 11%, *p* < 0.003).

In the further course of therapy, we also evaluated the prognostic relevance of an even stronger iFLC decrease of at least 75%. However, the achievement of this reduction at later times had no significant impact on prognosis.

The ten patients without light chain involvement had a similar outcome with a 48 months PFS (27% vs 37%; *p* = 0.88) and OS (62% vs 62%; *p* = 0.88) compared to the other patients.

## Discussion

In this monocentric retrospective study of BPV-treated newly diagnosed MM patients, we analyzed the prognostic potential of genetic and laboratory parameters. In particular, we found that early decline ≥ 50% in iFLC on day 8 resulted in favorable prognosis.

A synergistic effect of bendamustine and bortezomib was initially shown in myeloma cell lines (Zhang et al. 2008). In subsequent clinical studies, the combination of bendamustine with bortezomib and corticosteroids has proven to be very effective in the primary treatment of MM (Pönisch et al. 2014; Berdeja et al. 2017; Mateos et al. 2015; Knauf et al. 2020). Regarding VGPR or better, these clinical trials showed a rate of 53–68% success rate and an ORR of 77–91%. Our data also confirm that BPV is highly active in patients with newly diagnosed MM. The resulting ORR of 88% with 51% of patients achieving VGPR or better is favorable compared to responses reported for the other bendamustine/bortezomib combination regimens.

In the present study, we found no differences in PFS or OS between patients with light chain and IgG or IgA myeloma. This contrasts to the results of previous studies in which a less favorable clonal evolution with poorer prognosis was found in patients with light chain predominant secretory MM (Brioli et al. 2014; Avivi et al. 2017), Although our analysis is restricted by the limited number of patients, it is possible that the combination of bendamustine with bortezomib ameliorates the negative prognostic value of light chain predominance.

Ludwig et al. (2014) shows that the combination of bendamustine and bortezomib improves considerably the prognosis of patients with high-risk cytogenetic in relapsed/refractory MM. In our study, we also found PFS and OS to be comparable in newly diagnosed MM patients with or without high-risk cytogenetically aberrations.

In the present study, we confirm our previous observation that BPV is a highly effective therapy in patients with renal insufficiency (Pönisch et al. 2012, 2014). In our analysis, we observed a favorable renal response rate of 64%, comparable to those observed for other regimes containing novel agents (Dimopoulos et al. 2016). In 23 of 36 patients with stage 3–5 renal insufficiency, we found a rapid recovery of renal function during the first two BPV cycles, with three of seven dialysis-dependent patients reverting to independence. The combination of bortezomib with bendamustine and prednisone induced a rapid reduction in light chain production in the first few days of treatment, potentially preventing the development of irreversible renal failure (Pönisch et al. 2014; Knauf et al. 2020).

The majority of the patients we examined (89%) presented at diagnosis with abnormal iFLC, which is comparable to findings of other studies (range 70–100%) (Dispenzieri et al. 2009). The influence of the initial FLC values on prognosis has been examined previously in various settings. In the era of conventional chemotherapy, Dispenzieri et al. (2008) demonstrated that patients with sFLC values above 117 mg/L had significantly shorter PFS and OS. After the introduction of the new drugs especially bortezomib-containing combinations, the Italian study group of Tacchetti et al. (2016) found that PFS, but not OS was reduced in patients with sFLC levels above 100 mg/L. This contrasts to our observation that neither sFLC nor sFLCr showed an impact on PFS or OS. Under conventional chemotherapy, a highly pathological ratio of sFLC was associated with an unfavorable prognosis (Kyrtsonis et al. 2007; Snozek et al. 2008). In the current studies with modern therapy regimens, this negative impact of sFLCr on prognosis is no longer apparent (Lopez-Anglada et al. 2018). Our own results confirm this, suggesting that the high efficacy of bortezomib-containing therapies can overcome the poor prognosis caused by an unfavorable clonal evolution of light chain-predominant secretory MM.

Owing to the short half life of FLCs (2–6 h, compared with 3–25 days for intact immunoglobulins), monitoring the iFLC is attractive to derive markers of disease response (Pratt et al. 2006; Tosi et al. 2013; Yağcı et al. 2015). Various studies have investigated the prognostic significance of an iFLC reduction during the first treatment cycles. However, these studies are difficult to compare, because they employed different therapy regimens with heterogenous response kinetics. An additional issue is the heterogeneity of drug combinations considered in some of these studies (Kyrtsonis et al. 2007; Hansen et al. 2014; Yağcı et al. 2015). Although conventional chemotherapy alone usually requires at least six cycles to achieve maximum response, bortezomib-containing therapies only require 2–4 cycles. We hypothesize that the kinetics of light chain reduction show similar dynamics. Accordingly, a significant decrease of iFLC/sFLCr after two cycles was shown to be a prognostic factor for ORR (Hassoun et al. 2005) and OS (Dispenzieri et al. 2008; Yağcı et al. 2015) in conventional chemotherapy studies. In contrast, bortezomib containing therapies lead to a more rapid reduction of sFLC (van Rhee et al. 2007; Tessenow et al. 2017). In the present study, 45% of the patients treated with BPV reached a ≥ 50% reduction of the iFLC by day 8. PFS and CR/sCR rate for these patients was significantly better than that of patients with a lesser degree of iFLC reduction. However, OS was improved only slightly, and the effect did not reach statistical significance.

These results are in contrast with the observation of the Little Rock Group (van Rhee et al. 2007) that high initial levels of iFLC and a rapid reduction during treatment define an aggressive MM subtype with poor prognosis.

A limitation of the present study is that the relatively small number of cases. This could result in false negative findings. Moreover, due to the exploratory nature of these retrospective analysis, we refrained from multiple testing correction so that validation of our findings in independent studies is recommended. Finally, we specifically investigated the BPV regimen limiting generalizability of our findings to other therapy scenarios.

In summary, these results indicate that a rapid decrease in the iFLC on day 8 is a promising prognostic parameter for newly diagnosed MM patients under BPV treatment. However, reduction in iFLC at later time points during therapy showed no additive prognostic value. Validation of our findings in larger number of patients and under different induction therapies is required.
